# Antioxidant Capacity of Free Volatile Compounds from *Olea europaea* L. cv. Oblica Leaves Depending on the Vegetation Stage

**DOI:** 10.3390/antiox10111832

**Published:** 2021-11-18

**Authors:** Renata Jurišić Grubešić, Marija Nazlić, Tina Miletić, Elma Vuko, Nenad Vuletić, Ivica Ljubenkov, Valerija Dunkić

**Affiliations:** 1 Faculty of Medicine, University of Rijeka, Braće Branchetta 20, HR-51000 Rijeka, Croatia; renatajg@medri.uniri.hr; 2 Faculty of Science, University of Split, Ruđera Boškovića 33, HR-21000 Split, Croatia; mnazlic@pmfst.hr (M.N.); elma@pmfst.hr (E.V.); nenov@pmfst.hr (N.V.); iljubenk@pmfst.hr (I.L.); 3 Pharmacy “Vaše Zdravlje”, Put Kotlara 50, Zadar, HR-23000 Zadar, Croatia; tina.miletic@vasezdravlje.hr

**Keywords:** antioxidant capacity, docosane, *(E)-β*-damascenone, essential oil, gas chromatography–mass spectrometry (GC-MS), hydrosol, myristicin, olea leaves, oleic acid

## Abstract

Previous research on specialized metabolites of olive leaves has focused on the phenolic components and their biological role. The research in this article focuses on the metabolites that form free volatile compounds (FVCs). The composition of FVCs is divided into compounds isolated in the oil phase (essential oils; EO) and in the aqueous phase (hydrosols; Hy) from leaves of *Olea europaea* L. cultivar Oblica. Plant material was collected from the same olive tree over a six-month period, from December to May, and analyzed by gas chromatography–mass spectrometry (GC–MS). The compounds *β*-caryophyllene, α-humulene, *allo*-aromadendrene, docosane, hexadecanoic acid and oleic acid were identified in all EO study periods. In the Hy in all studied periods, the major compounds are α-pinene, *β*-ionone, myristicin, docosane, *1*-hexanol, oleic acid and *(E)-β*-damascenone. The differences in the qualitative composition of FVC are directly related to the phenological development of the leaves. Antioxidant capacity of the EOs and hydrosols was measured with two methods, ORAC and DPPH. Hydrosol extracts showed higher capacity than the EOs in all methods.

## 1. Introduction

*Olea europaea* L. (Oleaceae)—the olive—is cultivated in various parts of the world, with the Mediterranean region still being the largest growing region due to its climate of warm, dry summers and cold, rainy winters [1]. This plant is so popular because the Mediterranean diet is considered one of the healthiest diets in the world and olive oil is one of the most important dietaries of this diet [2]. 

Of the six subspecies of olive, only three are naturally distributed in the Mediterranean: subsp. *europaea*, subsp. *guanchica*, and subsp. *cerasiformis. Olea europaea* subsp. *europaea* is a subspecies considered drought resistant because it grows in an areas where it is frequently exposed to stress (e.g., lack of water), which is characteristic of the Mediterranean region [3]. It is divided into a wild form *O. europaea* subsp. *europaea* var. *sylvestris* and a cultivated form *O. europaea* subsp. *europaea* var. *europaea* [4]. The area under olive cultivation in Croatia is very large and the most important variety is “Oblica”, which is the subject of the present study [5,6]. 

Olive leaves are one of the by-products of olive cultivation and are available throughout the year. Since the leaf is a plant organ where most of the primary and specialized metabolism takes place, it is clear that it is an important source of bioactive components responsible for numerous pharmacological effects [7]. 

Although the fruit has been the most commonly used part of the olive since ancient times, the olive leaf has also been traditionally used for various purposes: orally to treat various intestinal disorders; the leaf was chewed to treat diseases of the oral cavity; in the form of a decoction, the leaf was used to treat diarrhea and urinary tract infections; a hot aqueous extract of fresh leaves was used orally to treat hypertension and stimulate urination, while a hot aqueous extract of dried leaves was used to treat asthma. Olive leaves have also been found to have a beneficial effect on the circulatory system, reducing muscle spasms in the intestines and relieving cardiac arrhythmias [8,9,10,11].

The bioactivity of olive leaves has traditionally been associated with their phenolic derivative content. Olive leaves contain five groups of phenolic compounds: oleuropeosides (oleuropein and verbascoside), flavones (luteolin-7-glucoside, apig-enin-7-glucoside, diosmetin-7-glucoside, luteolin and diosmetin), flavonols (rutin), flavan-3-ols (catechin) and substituted phenols (tyrosol, hydroxytyrosol, vanillin, vanillic acid and caffeic acid) [12,13]. Many researchers agree that oleuropein is the compound in olive leaf that is most responsible for its beneficial effects on health, as its hydrolysis products also exhibit significant pharmacological effects [14,15,16,17]. Oleuropein has several proven biological activities such as antioxidant, anti-inflammatory, anticancer, antiatherogenic, antimicrobial, antiviral, cardioprotective, anti-ischemic, neuroprotective and hypolipidemic activity [18]. It has a beneficial effect on age-related diseases, such as dementia. Other biological activities include lowering blood sugar levels, liver protection, stomach protection, weight loss and osteoporosis prevention [19]. It is also used in cosmetics as it protects against UVB rays, slows the aging process, and promotes wound healing and re-epithelialization [20,21]. Many phenolic compounds isolated from olive leaves can exert a pronounced antioxidant activity, which in turn can accelerate the healing process of ulcers [22]. Generally, the olive leaf has the highest antioxidant activity of all parts of the olive tree [11,23]. Olive leaf contains a greater amount and variety of polyphenols than extra virgin olive oil. Moreover, there are important structural differences between polyphenols from olive leaves compared to those from fruits that could have the more significant beneficial effect of olive leaf extract on human health [16]. 

These polyphenols components belong to the specialized metabolites as well as the terpenoid components, also described in olive leaves, including monoterpenes, sesquiterpenes, diterpenes and triterpenes [16,24]. These free volatile compounds (FVCs) have been best studied in olive fruit and are dominated by C5 and C6 compounds [25]. GC and gas chromatography–mass spectrometry (GC-MS) analysis of essential oils (EO) in olive leaves from Tunisia resulted in the identification of 32 compounds, of which monoterpene hydrocarbons accounted for 55.16% of the total EO and α-pinene (52.70%) was the main compound. In this study DPPH radical-scavenging activity of EO compounds was investigated [26]. Also, α-pinene was the main compound in olive leaves of Turkish variety [27]. Brazilian olive leaves contain a significant amount of polyunsaturated fatty acids [28]. Campeol et al. investigated the chemical composition of volatile fractions from leaves of three *Olea europaea* L. cultivars Leccino, Frantoio and Cipressino from Italy harvested in two different seasons by GC and GC-MS. The results showed a high content of aliphatic aldehydes and an increase in *(E)-2-*hexenal content from July to November [29]. Extracts obtained from wild olive leaves (*O. europaea* ssp. *sylvestris*) also showed important biological activity [30].

In previous study by Popović et al., EOs from leaves of *O. europaea* subsp. *europaea* of Croatian cultivars: Oblica, Lastovka, Leccino and Frantoio were investigated in the period of August, September and October. The results showed that Oblica cultivar EO was abundant in aldehydes, ketones and sesquiterpenes [6]. Despite the numerous biological effects of polyphenols contained in olive leaves, the hydrosols obtained from olive leaves have never been phytochemically analyzed, and we hypothesize that these harmless water extracts may be a readily available source of biologically active substances with antioxidant capacity. In addition, phytochemical analysis of essential oils and hydrosols over a continuous period of six months, as performed in this study, will provide information on the composition of olive volatiles in the lipophilic and aqueous fractions over a longer period of the year, as well as on the antioxidant activity of these extracts in different vegetative phases, from winter period to flowering phase. Our team decided to study FVCs during this period because this is the time of pruning of olive trees. The leaves obtained and their extracts could be a source of biologically active substances that could be used for the protection of food, natural cosmetic products and natural-based pharmaceuticals. Previous research on olives in relation to months has focused on temperature changes, which are important for the onset of flowering [28]. Our research focuses on the antioxidant capacity of free volatile compounds in the essential oils and hydrosols from the leaves of olive cv. Oblica. These studies have confirmed that, in addition to the already well-researched phenolic components, the free volatile compounds have a significant impact on the total antioxidant value of olive leaves.

## 2. Materials and Methods

### 2.1. Plant Material and Isolation of Free Volatile Compounds

Plant material for *Olea europaea* L cv. Oblica was collected from N 44°18′40.70″, E 15°16′27.23″ location in Croatia (Ražanac, Zadar). Voucher specimens were deposited in the Laboratory of Botany herbarium (HPMF-HR), Faculty of Science, University of Split, Croatia. The first sample was collected on 12 December 2019; the second sample on 6 January 2020; the third sample on 3 February 2020; the fourth sample on 14 March 2020; the fifth sample on 14 April 2020 and the sixth sample on 14 May 2020. Samples were air dried in a single layer and protected from direct sunlight, for then days.

Dried olive leaves (100 g) for each month were hydrodistilled for 3 h in a Clevenger-type apparatus (Šurlan, Medulin, Croatia). The extracts were collected in two phases in the inner tube of the Clevenger apparatus. The lipophilic fraction was extracted in pentane and diethyl ether (VWR, Radnor, PA, USA) and the hydrophilic volatile compounds were extracted in the water layer and stored in the refrigerator until analysis.

### 2.2. Extraction of Volatiles from Hydrosols

Volatiles dissolved in water fraction have been extracted with pentane/diethyl ether mixture to determine antioxidant capacity of hydrosols and compare it with EO antioxidant capacity. Each hydrosol (5 mL) was extracted with the mixture of 3 mL of pentane and 3 mL of diethyl ether in a separation funnel. The nonpolar layer was separated and dried over the anhydrous sodium sulphate. The solvent from the nonpolar layer was evaporated to calculate the exact concentration of hydrosol. The volatile compounds that remained were weighed and the concentration for each hydrosol sample was calculated and expressed in mg/mL of hydrosol.

### 2.3. Gas Chromatography, Gas Chromatography—Mass Spectrometry and Columns Conditions

Both phases, lipophilic and hydrophilic were analyzed by GC and GC-MS. GC was performed using model 3900; Varian Inc., Lake Forest, CA, USA equipped with a flame ionization detector (FID), a MS model 2100T; Varian Inc., Palo Alto, CA, USA. The chromatographic conditions were: FID detector temperature 300 °C, injector temperature 250 °C, the carrier gas was helium at 1 mL min^−1^. The MS conditions were: ion source temperature 200 °C, ionization voltage 70 eV, mass scan range 40–350 mass units [31,32].

Samples were analyzed on two columns: non-polar capillary column VF-5ms (30 m × 0.25 mm i.d., coating thickness 0.25 μm, Palo Alto, CA, USA) and a polar CP Wax 52 CB (30 m × 0.25 mm i.d., coating thickness 0.25 μm; Palo Alto, CA, USA) was equipped. The conditions for the columns were: VF-5ms (temperature 60 °C isothermal for 3 min, then increased to 246 °C at a rate of 3 °C min^−1^, and held isothermal for 25 min) and for the CP Wax 52 column (temperature 70 °C isothermal for 5 min, then increased to 240 °C at a rate of 3 °C min^−1^, and held isothermal for 25 min). 

### 2.4. Analyses of Free Volatile Compounds

The injected volume for lipophilic fractions were 2 μL and the split ratio was 1:20. For the hydrophilic fraction, injection was performed with a headspace injection needle and there was no split ratio (splitless mode). The 2 g of hydrosol was added to the glass bottle and sealed with a metal cap with septum. The headspace needle was injected into the glass bottle sealed with a metal cap with septum. The glass bottle was first placed in 40 °C water with the hydrosol sample and left there for 20 min without the needle to allow the volatile compounds to evaporate from the water. The needle was then injected and left there for 20 min to allow the volatile compounds to adsorb onto the resin needle. The injection needle was then inserted into a GC inlet and left there for 20 min [33]. Analyses was started after the 10 min of resorption in the liner and the needle was left in the liner for 10 min more during the analyses to ensure that all volatile compounds were reabsorbed from the resin into the injection liner.

The individual peaks for all samples were identified by comparing their retention indices of n-alkanes with those of authentic samples and literature [31]. The results for all samples were measured in three independent analyzes and expressed as a percentage (%, relative peak area) of each compound (Tables 2 and 3).

### 2.5. Antioxidant Capacity of Essential Oils and Hydrosols

#### 2.5.1. ORAC

The assay was performed in a Perkin–Elmer LS55 spectrofluorimeter (Perkin-Elmer, Leatherhead, UK), using 96-well white polystyrene microtiter plates (Porvair Sciences, Leatherhead, UK) following a method described by Fredotović et al. [34], with some adjustments based on different extracts. Hydrophilic assay was performed for hydrosols and lipophilic assay for EOs. Adjustments were made for the EO antioxidant assay. EOs were dissolved in acetone (10 mg in 1 mL acetone). The EO acetone dilutions were further dissolved 40× and 80× in the phosphate buffer prior to the experiments. Hydrosols were diluted 2× and 10× in the phosphate buffer prior to the experiments. All measurements were performed in triplicate according to the method described in Nazlić et al. [33].

#### 2.5.2. DPPH

The antioxidant capacity of the extracts was determined using the DPPH method already described by Mensor et al. and Payet et al. [35,36] and adapted to tested plant extracts. Plant extracts as described in the ORAC method were used (acetone-dissolved essential oils and absolute hydrosols) for the assay. An amount of 100 µL of methanol (Kemika, Zagreb, Croatia) and 200 µL of sample was pipetted into each well. Serial dilutions of samples were prepared by pipetting 100 µL from the first row with a multichannel pipette into the wells in the second row and so on to the last row, where 100 µL of the solution was ejected after mixing. In the first column, in 96-well plates, a blank sample was always added. For EOs, the acetone and methanolic solution were used as blank and for hydrosols, water and methanolic solution were used as blank. The calculation and presentation of the results were performed according to the method described in the previous research by Nazlić et al. [33].

### 2.6. Statistical Analyses

Statistical analysis was performed in GraphPad Prism Version 9 (GraphPad Software, San Diego, CA, USA). All data are expressed as mean ± SD (*n* ≥ 3). The statistical significance for antioxidant activity was assessed by 2-way ANOVA followed by Šídák’s multiple comparisons test, *p* < 0.05. These statistical tests were performed separately for lipophilic (essential oils) and hydrophilic fractions (hydrosols). 

## 3. Results and Discussion

### 3.1. Pretreatment and Isolation of Free Volatile Compounds from Olive Leaves

During a period of six months the leaves of *Olea europaea* L. cultivar Oblica were collected. Oblica is the most widespread native variety in the Mediterranean part of Croatia. It tolerates stressful conditions well and develops a medium-sized, lush tree with a round crown and numerous grey-olive leaves with non-glandular trichomes [5]. Olive trichomes are transcriptionally active and represent a tissue involved in developmental processes and specialized metabolism [37]. Leaves for this study were collected from December to May (Table 1), the period after olive harvest, from winter dormancy to flowering. The differences in the composition of free volatiles under different climatic conditions were studied, while the geographical location and soil were the same, since leaves were collected from the same olive tree. The collected olive leaves were air dried. Several studies have reported different types of pretreatment of collected olive leaves before extraction. Pretreatment is important for the isolation of the target substances, therefore olive leaves are prepared under different conditions (air, water and temperature) for extraction [9].

Various extracts have been prepared from olive leaves: ethanol/water extracts obtained with microwaves and different combinations of extractions with ethanol, butanol/ethyl acetate fractions, hexane, chloroform/water fractions and water/ethanol/citric acid [38]. In this study, two phases of extracts were collected in a central measuring tube of the Clevenger apparatus. Pentane and diethyl ether were used as solvents of EOs. FVCs were isolated in the lower aqueous layer and in the upper pentane/diethyl ether layer. The common name for the volatiles soluble in pentane is essential oils and those soluble in water are hydrosols. Table 1 shows the masses of the isolated volatile compounds of the EOs and hydrosols, and the yields were calculated based on the dry plant material used for the extractions. It can be seen that a greater amount of volatile compounds is isolated in the hydrosols which could be the reason for the higher antioxidant capacity of these extracts compared to the EOs.

### 3.2. GC and GC-MS Identification of Free Volatile Compounds from Olive Leaves

The volatile compounds in the essential oil and hydrosols of *O. europaea* cv. Oblica were analyzed by GC and GC-MS and are listed in Table 2 and Table 3 in the order of elution from the non-polar column.

#### 3.2.1. Composition of Essential Oil

Table 2 shows the composition and percentage of identified FVCs in leaves of *O. europaea* cv. Oblica analyzed for six months. The total oil content was 0.1% for December to February, 0.04% in March, 0.2% in April and 0.08% in May (Table 1), based on the dry weight of the samples. These differences in percentage utilization can be explained by the differences in weathering and vegetation development of the olives. The winter months are the dormant period of the olives, and the total percentage of oil content is constant. Phenological growth of new olive leaves begins in March and lasts until November, but the time at which each growth stage is reached varies depending on the variety or year [32]. In April, a total oil content of 0.2% is formed due to the high metabolic activity in the leaves. The month of May is the time of budding and it is physiologically understandable that the metabolic products are directed from the leaves to the new vegetative shoots.

Differences were not only observed in the percentage of total oil, but also in the composition of the FVCs, as shown in Table 2. In the composition of identified volatile metabolites of olive leaves in December, the most abundant compounds are myristicin (33.02%), caryophyllene oxide (17.13%) and oleic acid (8.11%). These components are most abundant in January with a similar percentage of identification as in December (myristicin 27.85%, caryophyllene oxide 21.27% and oleic acid 6.74%). In the composition of the EO isolated in February, linalyl acetate (11.28%), endo-fenchyl acetate (12.75%), α-cubebene (10.54%) are the main components. These esters and sesquiterpene hydrocarbon were identified only in February, they were not detected in the other five months. According to Dursun et al., (2017) α-cubebene is a major compound collected in December of olive leaves from Hatay province (Turkey). In this study it was identified only in February with a value of 10.54% [39]. Linalool was identified in February with a high value of 9.35%, while in December it was detected only with a value of less than one percent. The isolates from March are rich in docosane (40.12%), *allo*-aromadendrene (20.94%) and *β*-caryophyllene (10.45%). These compounds were identified in all months with the highest yield in March. The compound *(E)-β-*damascenone was also identified in March with a high percentage of 10.45%, having previously been identified in January with 0.52 %. In April, the value of (E)-β-damascenone fell to 5.02%, and the dominant compounds were *β*-ionone (20.48%), α-ionone (18.56%), and *α*-humulene (18.53%). The compound *β*-ionone is formed as a cleavage product of β-carotene and it has a significant ecological role in attracting insects or as a repellent, and a significant biomedical role: antibacterial, fungicidal, anticancer and other benefits to human health [40]. The compound *α*-humulene (14.75%) was significantly present in May, followed by *(E)-β-*damascenone (14.55%). 

Overall, the compounds *β*-caryophyllene (0.39–10.45%), α-humulene (0.72–18.53%), *allo*-aromadendrene (1.44–20.94%), docosane (3.46–40.12%), hexadecanoic acid (0.75–9.93%) and oleic acid (3.36–8.94%) were identified in all the study periods (Table 2). The *β*-caryophyllene and *(E)-β-*damascenone were identified of March, April, August and November from Tunisian olive [41]. The *(E)-β-*damascenone was a one of major compound in Croatian cv. Oblica from period August, September and October [6] so it can be concluded that this compound is synthesized throughout the year.

#### 3.2.2. Composition of Hydrosol

Free volatile compounds of olive leaves identified from the aqueous part of the extract in all study months are α-pinene (1.36–7.45%), β-ionone (0.35–40.35%), myristicin (1.15–35.57%), docosane (0.46–4.89%), 1-hexanol (0.12–2.51%), oleic acid (1.18–12.57%) and (E)-β-damascenone (6.58–19.12%) (Table 3). Docosane and oleic acid were also identified in all months of the oil phase (Table 2). Myristicin is the most abundant compound in December Hy phase and belongs to the group of apiols known for their synergistic effects. It is used as an antidepressant, anti-inflammatory, UV-B protectant, insecticide and antioxidant [42,43].

The following compounds were identified in the hydrosols in only one month of the study (with a percentage higher than 2%), namely: bicyclogermacrene was identified in March with a high percentage of 39.54% as well as n-dodecanol identified with 5.16%; n-decanal (3.98%) and germacrene D (4.79%) are compounds identified only in January; endo-fenchyl acetate (2.75%) in February; α-thujene (7.12%) in April; α-copaene (3.55%) in May. The compound linalyl acetate was identified in February and March with a percentage slightly less than 10% for both months. 

### 3.3. Antioxidant Capacity

Olive leaves are the sites of synthesis of primary and secondary (specialized) metabolites with phenolic compounds being the most investigated category of specialized metabolites in the terms of their composition in the olive leaves and their biological activity [44]. Papoti et al. investigated antioxidant capacity of the phenolic compounds from the Greek olive tree cultivars [45]. They found that methanol and ethanol extracts showed almost identical DPPH capacity. Ferreira et al. investigated antioxidant capacity of phenolic compounds from the olive leaves and they found that copper formulation sprayed on the olive leaves affect their phenolic composition and therefore DPPH capacity [46]. Highest antioxidant capacity showed leaves that were not sprayed with any copper formulations and their values were similar to those of the reference compounds. Kiritsakis et al. in their research of antioxidant capacity of phenolic extracts from Greek cultivars of olive leaves concluded that part of the capacity comes from other unidentified compounds [47]. These unidentified compounds could be terpenes and other volatile compounds which are the subject of this study.

**Figure 1:**
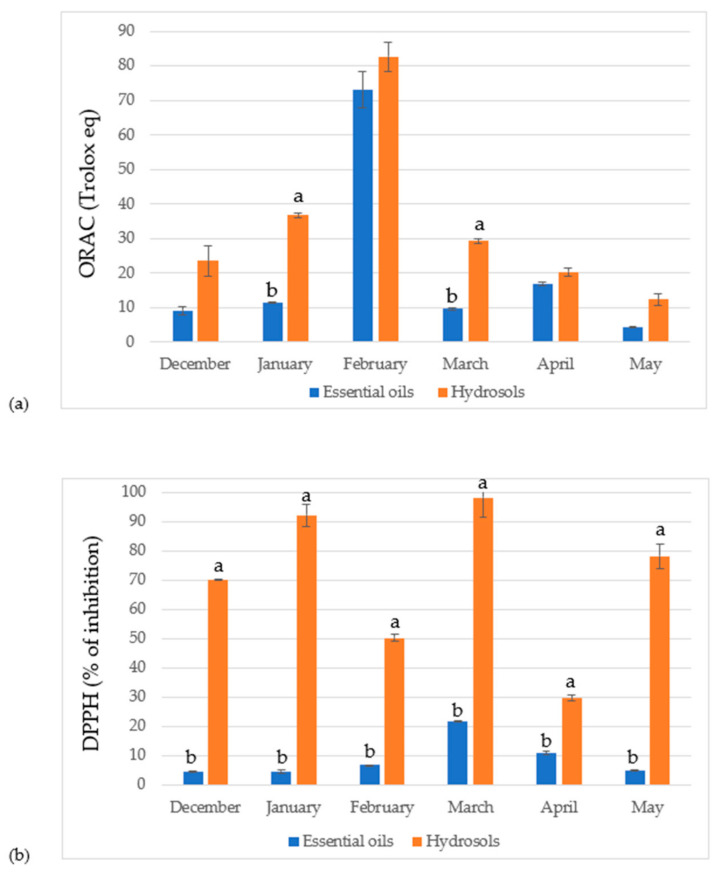
Antioxidant capacity determined by (**a**) ORAC assay (µmol TE/g, mean values) and (**b**) DPPH assay (percentage of inhibition, mean values) in olive leaves harvested in different periods from Oblica cultivar. Error bars indicate SD of the means (*n* = 3); significant differences between EOs and hydrosols were determined using multiple t-test. ^a,b^ Mean values with different superscript letters indicate a statistically significant difference between EOs and hydrosols for each month (*p* < 0.05).

In the Table 4 results for the antioxidant capacity of the free volatile compound extracts measured with two methods, ORAC and DPPH, were reported. In the ORAC method, EO and hydrosol from the February sample had the highest capacity (73.12 ± 5.24 and 82.66 ± 4.37 µmol/g, respectively) with the hydrosol having slightly higher capacity than the EO. In the DPPH method, EO and hydrosol from the March sample showed the highest capacity (IC_50_ 23.58 ± 0.18 and 4.16 ± 0.28 mg/mL, respectively) with hydrosol having approximately five times higher antioxidant capacity than the EO. This is in agreement with another study by Blasi et al. in which they studied seasonal variations in antioxidant capacity of phenolic compounds. They reported the highest antioxidant activity in March [48]. In general, hydrosols from all studied leaf stages of vegetation have higher antioxidant capacity than EOs (Figure 1). This is probably due to the higher content of volatile compounds isolated in hydrosols when compared to the amounts in the EOs (Table 1). Also, when comparing the identified compounds in the EOs and hydrosols it can be seen that hydrosols contain more identified compounds belonging to monoterpene hydrocarbons. Ruberto et al. found that compounds from this category showed significant antioxidant capacity [49]. The reason for the higher antioxidant capacity of the olive leaves collected in February could be due to higher percentage of linalool when compared to other months (9.35 ± 0.01 %). Baschieri et al. studied antioxidant activity of some common non-phenolic compounds found in EOs and their study showed that linalool can contribute to the antioxidant capacity of natural essential oils [50]. The essential oil from March have higher percentage of *β*-caryophyllene that is known for its antioxidant that appears to be similar to the activity of the ascorbic acid [51]. Hydrosols from March have high percentage of bicyclogermacrene (39.54 ± 0.01 %) when comparing to other months and this could potentially be the reason for the highest activity when measured by DPPH method. In a study by da Silva et al., where they compared antioxidant activity of the EO from *Endlicheria arenosa* leaves and twigs, leaves had high content of bicyclogermacrene and this EO shoed higher activity than the EO from twigs [52]. Hydrosols from February and March have high relative amount of *β*-Ionone (40.35 ± 0.01 %, 42.87 ± 0.01 %, respectively) which could also be the reason for higher antioxidant capacity in these months.

Viuda-Martos et al. tested the antioxidant activity of EOs from five spice plants that are commonly used in the Mediterranean and comparing their results with current study it can be noticed that all samples of hydrosols have higher activity than rosemary EO (17 mg/mL) and the hydrosol from March has similar activity to the activity of sage EO (4.20 mg/mL) [53]. Many studies have confirmed that olive leaves are a potential source of biologically active by-products of olive oil production generated during pruning [44]. The results show that the antioxidant capacity of volatiles was highest from in February and March. The fact that olive leaf pruning is very important at this stage of vegetation suggests the potential use of pruned leaves as an antioxidant source that can be used in pharmacy and food protection. Future research on these extracts may address their antimicrobial, antiproliferative and antiphytoviral potential.

## 4. Conclusions

The results of this study show that oleic acid and docosane are components identified in the hydrophilic and lipophilic phases, i.e., essential oil and hydrosol, extracted from the leaves of *Olea europaea* cv. Oblica in all study periods. Other significant FVCs greater than 10% identified in at least one month of research are: *β*-cubebene, *β*-caryophyllene, *α*-humulene, *allo*-aromadendrene, bicyclogermacrene, caryophyllene oxide, myristicin, *(E)-β-*damascenone, *α*-ionone, *β*-ionone, endo-fenchyl acetate and linalyl acetate. Despite the numerous biological effects of olive leaf polyphenols, this study showed that leaf essential oils also have a significant antioxidant capacity, reaching their maximum in February and March. For the first time, the hydrosols of olive leaves were phytochemically analyzed and the data showed that these harmless water extracts could also be a readily available source of biologically active substances with pronounced antioxidant activity. Volatile compounds showed highest antioxidant capacity in February and March, when olive trees begin new growing season and become more metabolically active. Overall, higher levels of volatile compounds were found in hydrosols, and they also exhibited higher antioxidant capacity than the essential oils in both methods used. This study showed that the previously described phenolic compounds are not the only specialized metabolites involved in the antioxidant capacity of olive leaves. Free volatile compounds contribute to the total antioxidant capacity of olive leaves.

## Figures and Tables

**Table 1:** Yield of obtained volatile compounds from essential oils (EOs) and hydrosols (Hy) of *Olea europaea* cv. Oblica.

Month	Mass of EO (mg)	Yield of EO (%)	Mass of Volatiles from Hy (mg)	Yield of Volatiles from Hy (%)
December	100	0.1	204	0.20
January	100	0.1	134	0.13
February	100	0.1	162	0.16
March	40	0.04	221	0.22
April	200	0.2	191	0.19
May	80	0.08	225	0.23

**Table 2:** Chemical composition of the essential oil of *Olea europaea* L. cv. Oblica from six months.

	Month of Collection/ Yield in %/ Yield in % ± SD							
Component	RI^1^	RI^2^	December (0.1%)	January (0.1%)	February (0.1%)	March (0.04%)	April (0.2%)	May (0.08%)
Monoterpene hydrocarbons			1.9	0.38	0.28	-	-	-
*α*-Thujene	924	1012	0.62 ± 0.01	-	-	-	-	-
*α*-Pinene *	935	1017	0.32 ± 0.01	0.38 ± 0.01	-	-	-	-
Myrcene	988	1160	0.75 ± 0.01	-	-	-	-	-
*β*-Thujone	1121	1451	0.21 ± 0.05	-	0.28± 0.01	-	-	-
Oxygenated monoterpenes			1.41	-	9.9	-	-	-
Linalool *	1099	1548	0.88 ± 0.01	-	9.35 ± 0.01	-	-	-
Borneol *	1176	1699	0.21 ± 0.04	-	0.17 ± 0.03	-	-	-
Terpinen-4-ol	1184	1601	0.32 ± 0.01	-	0.38 ± 0.01	-	-	-
Sesquiterpene hydrocarbons			10.48	16.58	24.32	32.37	30.86	40.57
α-Cubebene	1345	1458	-	-	10.54± 0.01	-	-	-
*α*-Copaene	1374	1484	-	3.29 ± 0.03	-	-	-	7.79 ± 0.01
*β*-Cubebene	1387	1540	-	-	1.34 ± 0.01	-	2.64 ± 0.01	8.99 ± 0.01
*β*-Elemene	1389	1593	1.64 ± 0.01	0.63 ± 0.01	-	-	-	0.66 ± 0.07
*β*-Caryophyllene *	1424	1585	2.82 ± 0.01	2.87 ± 0.02	1.86 ± 0.01	10.45 ± 0.01	0.39 ± 0.06	1.26 ± 0.01
*β*-Copaene	1429	1576	0.27 ± 0.05	3.26 ± 0.02	2.25 ± 0.01	-	2.95 ± 0.01	2.38 ± 0.01
*(Z)-β*-Farnesene	1454	1639	0.24 ± 0.1	0.29 ± 0.01	-	-	-	-
*α*-Humulene	1456	1654	0.75 ± 0.01	0.78 ± 0.01	0.78 ± 0.01	0.72 ± 0.01	18.53 ± 0.01	14.75 ± 0.01
*allo*-Aromadendrene	1465	1662	2.92 ± 0.05	2.98 ± 0.02	1.44 ± 0.02	20.94 ± 0.01	5.98 ± 0.01	4.74 ± 0.01
Germacrene D	1481	1692	0.46 ± 0.01	0.99 ± 0.03	4.76 ± 0.01	0.26 ± 0.02	0.37 ± 0.01	-
*β*-Bisabolene	1494	1729	0.63 ± 0.01	0.75 ± 0.01	0.64 ± 0.07	-	-	-
*δ*-Cadinene	1517	1745	0.75 ± 0.01	0.74 ± 0.01	0.71 ± 0.01	-	-	-
Oxygenated sesquiterpenes			21.91	22.38	1.34	-	0.68	7.03
Spathulenol	1577	2101	0.32 ± 0.01	0.35 ± 0.05	0.38 ± 0.05	-	-	-
Caryophyllene oxide *	1581	1955	17.13 ± 0.03	21.27 ± 0.01	0.96 ± 0.01	-	0.68 ± 0.01	-
α-Cadinol	1655	2208	4.46 ± 0.01	0.76 ± 0.03	-	-	-	7.03 ± 0.01
Phenolic compounds			33.02	27.85	4.18	2.03	-	2.95
Eugenol *	1370	2175	-	-	-	0.91 ± 0.1		
Myristicin	1520	2260	33.02 ± 0.01	27.85 ± 0.01	4.18 ± 0.01	1.12 ± 0.01	-	2.95± 0.01
Hydrocarbons			13.03	10.72	19.25	42.81	6.9	8.58
Heneicosane *	2100	2100	0.23 ± 0.01	0.24 ± 0.02	0.26 ± 0.01	-	0.27 ± 0.01	-
Docosane *	2200	2200	5.21 ± 0.01	4.54 ± 0.03	7.03 ± 0.01	40.12± 0.01	6.63 ± 0.01	3.46 ± 0.01
Tricosane *	2300	2300	0.12 ± 0.03	0.14 ± 0.01	0.15 ± 0.01	0.53± 0.01	-	0.44 ± 0.01
Tetracosane *	2400	2400	-	0.48 ± 0.02	3.46 ± 0.06	1.24± 0.07	-	4.68 ± 0.03
Pentacosane *	2500	2500	1.52 ± 0.01	1.56 ± 0.03	1.54 ± 0.01	-	-	-
Hexacosane *	2600	2600	4.63 ± 0.01	2.94± 0.02	2.96 ± 0.01	-	-	-
Heptacosane *	2700	2700	1.32 ± 0.01	0.82 ± 0.01	3.85 ± 0.01	0.92± 0.01	-	-
Alcohols			-	0.93	-	-	-	0.64
n-Decanol	1266	1714	-	0.93 ± 0.01	-	-	-	-
n-Dodecanol	1469	1960	-	-	-	^-^	-	0.64± 0.01
Acids			11.32	9.52	8.41	6.1	13.59	9.69
Hexadecanoic acid	1959	2913	3.21 ± 0.01	2.78 ± 0.03	2.94± 0.01	1.18± 0.01	9.93 ± 0.01	0.75± 0.01
Oleic acid	2139	-	8.11 ± 0.02	6.74 ± 0.02	5.47± 0.01	4.92± 0.01	3.66 ± 0.01	8.94± 0.01
Ketones			-	8.02	1.24	11.97	44.06	23.58
(E)-β-Damascenone	1384	1819	-	0.52 ± 0.05	-	10.45 ± 0.01	5.02 ± 0.01	14.55 ± 0.01
α-Ionone	1427	1843	-	5.32 ± 0.01	-	1.52 ± 0.02	18.56 ± 0.01	0.75 ± 0.01
β-Ionone	1487	1935	-	2.18 ± 0.01	1.24 ± 0.01	-	20.48 ± 0.01	8.28 ± 0.01
Esters			-	-	24.03	-	-	-
endo-Fenchyl acetate	1218	1465	-	-	12.75 ± 0.01	-	-	-
Linalyl acetate	1252	1553	-	-	11.28 ± 0.01	-	-	-
Total identification (%)			93.07	96.38	92.95	95.28	96.09	93.04

Retention indices (RIs) were determined relative to a series of n-alkanes (C8–C40) on capillary columns VF5-ms (RI^1^) and CPWax 52 (RI^2^).Method of identification: RI, comparison of RIs with those in a self-generated library reported in the literature [31] and/or with authentic samples; comparison of mass spectra with those in the NIST02 and Wiley 9 mass spectral libraries [32]; * co-injection with reference compounds; -, not identified; SD, standard deviation of triplicate analysis.

**Table 3:** Chemical composition of the hydrosols of *Olea europaea* L. cv. Oblica from six months.

	Month of Collection/ Yield in % ± SD							
Component	RI^1^	RI^2^	December	January	February	March	April	May
Monoterpene hydrocarbons			4.16	2.65	1.36	1.79	13.35	7.45
*α*-Thujene	924	1012	-	-	-	-	7.12 ± 0.01	-
*α*-Pinene *	935	1017	2.68 ± 0.01	2.65 ± 0.01	1.36 ± 0.01	1.79 ± 0.01	6.23 ± 0.01	7.45 ± 0.01
Myrcene	988	1160	0.87 ± 0.01	-	-	-	-	-
*β*-Thujone	1121	1451	0.61 ± 0.01	-	-	-	-	-
Oxygenated monoterpenes			2.9	-	0.38	-	7.18	2.78
Linalool *	1099	1548	-	-	-	-	-	0.97 ± 0.01
Borneol *	1176	1699	-	-	-	-	1.27 ± 0.01	-
Terpinen-4-ol	1184	1601	-	-	-	-	0.79 ± 0.01	-
α-Terpineol	1186	1690	2.18 ± 0.01	-	-	-	3.66 ± 0.01	0.67 ± 0.07
Myrtenol	1197	1782	0.72 ± 0.01	-	0.38 ± 0.01	-	1.46 ± 0.01	1.14 ± 0.01
Sesquiterpene hydrocarbons			3.98	7.26	1.31	41.97	3.31	9.71
α-Copaene	1374	1484	-	-	-	-	-	3.55 ± 0.01
β-Elemene	1389	1593	1.16 ± 0.05	-	-	1.17 ± 0.01	-	-
β-Caryophyllene *	1424	1585	2.82 ± 0.01	1.38 ± 0.01	1.31 ± 0.01	1.26 ± 0.01	-	-
β-Copaene	1429	1576	-	-	-	-	-	0.86 ± 0.01
(Z)-β-Farnesene	1454	1639	-	-	-	-	1.24 ± 0.01	-
α-Humulene	1456	1654	-	-	-	-	2.07 ± 0.01	4.54 ± 0.01
allo-Aromadendrene	1465	1662	-	0.38 ± 0.03	-	-	-	-
Germacrene D	1481	1692	-	4.79 ± 0.01	-	-	-	-
β-Bisabolene	1494	1729	-	-	-	-	-	0.76 ± 0.01
Bicyclogermacrene	1500	1718	-	-	-	39.54 ± 0.01	-	-
δ-Cadinene	1517	1745	-	0.71 ± 0.01	-	-	-	-
Oxygenated sesquiterpenes			2.71	-	-	-	2.39	1.92
Spathulenol	1577	2101	0.47 ± 0.01	-	-	-	0.42 ± 0.01	-
Caryophyllene oxide *	1581	1955	0.96 ± 0.02	-	-	-	1.97 ± 0.01	0.67 ± 0.01
α-Cadinol	1655	2208	1.28 ± 0.05	-	-	-	-	1.25 ± 0.01
Phenolic compounds			43.05	2.17	3.36	6.26	10.33	11.75
Eugenol *	1370	2175	7.48 ± 0.01	1.02 ± 0.01	-	-	-	-
Myristicin	1520	2260	35.57 ± 0.01	1.15 ± 0.01	3.36 ± 0.01	6.26 ± 0.01	10.33 ± 0.01	11.75 ± 0.01
Hydrocarbons			0.46	2.97	3.27	3.57	6.01	3.67
Heneicosane *	2100	2100	-	-	-	-	-	0.32 ± 0.01
Docosane *	2200	2200	0.46 ± 0.01	0.94 ± 0.01	3.27 ± 0.01	3.31 ± 0.01	4.89 ± 0.01	1.15 ± 0.01
Tricosane *	2300	2300	-	0.35 ± 0.03	-	-	0.46 ± 0.01	0.94 ± 0.07
Tetracosane *	2400	2400	-	0.67 ± 0.03	-	-	0.66 ± 0.01	1.26 ± 0.01
Pentacosane *	2500	2500	-	0.46 ± 0.01	-	-	-	-
Hexacosane *	2600	2600	-	0.55 ± 0.02	-	-	-	-
Heptacosane *	2700	2700	-	-	-	0.26 ± 0.01	-	-
Alcohols			0.97	19.89	1.54	6.9	2.51	0.12
1-Hexanol	863	1350	0.97 ± 0.07	1.23 ± 0.01	1.22 ± 0.01	1.47 ± 0.01	2.51 ± 0.01	0.12 ± 0.01
n-Decanol	1266	1714	-	18.66 ± 0.01	0.32 ± 0.01	0.27 ± 0.01	-	-
n-Dodecanol	1469	1960	-	-	-	5.16 ± 0.01	-	-
Acids			3.15	2.67	12.82	11.12	9.69	7.02
Hexadecanoic acid	1959	2913	1.97 ± 0.01	-	0.25 ± 0.04	-	2.71 ± 0.01	4.67 ± 0.01
Oleic acid	2139	-	1.18 ± 0.05	2.67 ± 0.01	12.57 ± 0.01	11.12 ± 0.01	6.98 ± 0.01	2.35 ± 0.02
Aldehydes			-	3.98	4.67	3.76	-	-
n-Nonanal	1100	1390	-	-	1.28 ± 0.03	3.76 ± 0.01	-	-
(E,Z)-2,6-Nonadienal	1150	1582	-	-	3.39 ± 0.01	-	-	-
n-Decanal	1201	1472	-	3.98 ± 0.01	-	-	-	-
Ketones			32.33	50.99	51.65	7.59	41.1	51.27
(E)-β-Damascenone	1384	1819	19.12 ± 0.01	10.64 ± 0.01	8.78 ± 0.01	6.58 ± 0.01	16.37 ± 0.01	16.89 ± 0.01
α-Ionone	1427	1843	3.28 ± 0.01	-	-	0.66 ± 0.01	8.76 ± 0.02	9.05 ± 0.01
β-Ionone	1487	1935	9.93 ± 0.01	40.35 ± 0.01	42.87 ± 0.01	0.35 ± 0.01	15.97 ± 0.01	25.33 ± 0.01
Esters			-	-	12.41	9.91	-	-
endo-Fenchyl acetate	1218	1465	-	-	2.75 ± 0.02	-	-	-
Linalyl acetate	1252	1553	-	-	9.66 ± 0.01	9.91 ± 0.01	-	-
Total identification (%)			93.71	92.58	92.77	92.87	95.87	95.69

Retention indices (RIs) were determined relative to a series of n-alkanes (C8–C40) on capillary columns VF5-ms (RI^1^) and CPWax 52 (RI^2^).Method of identification: RI, comparison of RIs with those in a self-generated library reported in the literature [31] and/or with authentic samples; comparison of mass spectra with those in the NIST02 and Wiley 9 mass spectral libraries [32]; * co-injection with reference compounds; -, not identified; SD, standard deviation of triplicate analysis.

**Table 4:** Antioxidant capacity of the EOs and hydrosols of *Olea europaea* L. cv. Oblica over the period of six months.

**Essential Oils**						
**Antioxidant Assay**	**December**	**January**	**February**	**March**	**April**	**May**
ORAC (Trolox eq)	9.28 ± 1.19 ^b^	11.44 ± 0.08 ^b^	73.12 ± 5.24 ^a^	9.66 ± 0.24 ^b^	16.93 ± 0.43 ^b^	4.43 ± 0.16 ^b^
DPPH (% inhibition)	4.64 ± 0.03 ^b^	4.48 ± 0.52 ^b^	6.72 ± 0.14 ^b^	21.71 ± 0.25 ^a^	10.73 ± 0.57 ^ab^	4.95 ± 0.16 ^b^
DPPH (IC 50)	127.71 ± 3.38 ^d^	130.71 ± 18.34 ^d^	78.25 ± 0.90 ^b^	23.58 ± 0.18 ^a^	86.15 ± 3.05 ^b^	105.18 ± 5.71 ^c^
**Hydrosols**						
**Antioxidant Assay**	**December**	**January**	**February**	**March**	**April**	**May**
ORAC (Trolox eq)	23.62 ± 4.40 ^c^	36.83 ± 0.66 ^b^	82.66 ± 4.37 ^a^	29.28 ± 0.76 ^bc^	20.31 ± 1.30 ^c^	12.39 ± 1.71^d^
DPPH (% inhibition)	69.99 ± 2.62 ^c^	92.04 ± 3.83 ^a^	50.19 ± 1.33 ^d^	98.06 ± 6.52 ^a^	29.56 ± 1.06 ^e^	78.20 ± 4.16 ^b^
DPPH (IC 50)	9.01 ± 0.33 ^a^	5.41 ± 0.23 ^a^	9.96 ± 0.26 ^ab^	4.16 ± 0.28 ^a^	16.91 ± 0.61 ^b^	6.39 ± 0.34 ^a^

ORAC, oxygen radical absorbance capacity, results for EOs expressed as µmol of Trolox equivalents (TE) per g of EO (10 mg/mL) and for hydrosols as µmol of Trolox equivalents (TE) per g of the total (undiluted) tested hydrosol sample (10 mg volatiles/mL of hydrosol); DPPH, percentage (%) of inhibition was calculated for the concentrations of EOs and hydrosols of 10 mg/mL; IC50 expressed in mg/mL; SD = standard deviation of triplicate analysis; significant differences were determined using 2-way ANOVA followed by Šídák’s multiple comparisons test. ^a,b,c,d,e^ Mean values in the same row with different superscript letters indicate a statistically significant difference between data from six months (*p* < 0.05).

## Data Availability

The data is contained within the article.
